# Veterinary Regenerative Medicine for Musculoskeletal Disorders: Can Mesenchymal Stem/Stromal Cells and Their Secretome Be the New Frontier?

**DOI:** 10.3390/cells9061453

**Published:** 2020-06-11

**Authors:** Michela Mocchi, Silvia Dotti, Maurizio Del Bue, Riccardo Villa, Elia Bari, Sara Perteghella, Maria Luisa Torre, Stefano Grolli

**Affiliations:** 1 Department of Drug Sciences, University of Pavia, 27100 Pavia, Italy; michela.mocchi@unipv.it (M.M.); elia.bari@unipv.it (E.B.); sara.perteghella@unipv.it (S.P.); 2 Istituto Zooprofilattico Sperimentale della Lombardia e dell’Emilia Romagna, 25124 Brescia, Italy; silvia.dotti@izsler.it (S.D.); riccardo.villa@izsler.it (R.V.); 3 Freelance Veterinary Medical Doctor, 43121 Parma, Italy; mauriziodelbue@gmail.com; 4 PharmaExceed S.r.l., 27100 Pavia, Italy; 5 Department of Veterinary Medical Science, University of Parma, 43121 Parma, Italy; stefano.grolli@unipr.it

**Keywords:** mesenchymal stem cells, regenerative medicine, veterinary, secretome, extracellular vesicles, microvesicles, exosomes

## Abstract

Regenerative medicine aims to restore the normal function of diseased or damaged cells, tissues, and organs using a set of different approaches, including cell-based therapies. In the veterinary field, regenerative medicine is strongly related to the use of mesenchymal stromal cells (MSCs), which belong to the body repair system and are defined as multipotent progenitor cells, able to self-replicate and to differentiate into different cell types. This review aims to take stock of what is known about the MSCs and their use in the veterinary medicine focusing on clinical reports on dogs and horses in musculoskeletal diseases, a research field extensively reported in the literature data. Finally, a perspective regarding the use of the secretome and/or extracellular vesicles (EVs) in the veterinary field to replace parental MSCs is provided. The pharmaceuticalization of EVs is wished due to the realization of a Good Manufacturing Practice (GMP product suitable for clinical trials.

## 1. Introduction

In recent years, the focus of medical science has shifted from repair to regeneration. Regenerative medicine aims to recover the normal function of diseased or damaged cells, tissues, and organs using a set of different approaches, including cell-based therapies, able to stimulate and coordinate the processes headed to biological restoration. Regenerative medicine stands out as a top research interest area in medical fields, including both human and veterinary medicine.

Regenerative medicine is strongly related to the use of mesenchymal stromal cells (MSCs), whose safety and efficacy are now considered well established for humans [1,2]. In veterinary medicine, the reality is much diversified. Above all, there is enormous variability in terms of anatomy and physiology among the animal species in which regenerative medicine can be applied (mainly horses and dogs), with consequences on stem cells biology, mechanisms of action, and several aspects on their application, such as minor/major inflammation responses after MSCs administration in vivo. Moreover, sometimes, even between breeds, there is a large variability related to the predisposition to a certain type of pathologies than others (for example, an impact factor could be the animal lifestyle, their weight and size, or their genetic background).

In this scenario, finding a common thread to describe the state of the art of veterinary regenerative medicine is not easy because the landscape of clinical studies is quite limited (http://www.clinicaltrials.gov, last access 25 March 2020); nevertheless new knowledge is rapidly accumulating as a result of both induced animal disease models used in human pre-clinical studies, and a more rigorous approach in the designing of clinical trials based on naturally occurring diseases. This review aims to take stock of what is known about the MSCs and their use in the veterinary field. Aware that translating clinical results from one species to another is not so easy and often not realistically possible, it has been decided to focus and limit the research field on what has been more extensively reported in the literature data. Nowadays, the therapeutic application of MSCs seems to have the most promising results in musculoskeletal diseases, based on several works published in the last few years. Dogs and horses are the most actively studied species, as it results from the evaluation of the literature, thus they will be considered more in detail. Finally, a perspective regarding the use of the secretome and/or extracellular vesicles (EVs) in the veterinary field, instead of parental MSCs, is provided.

## 2. Mesenchymal Stromal Cells and Veterinary Regenerative Medicine: Main Features, Sources, Isolation, and Cryopreservation Procedures

MSCs are defined as multipotent progenitor cells able to self-replicate and differentiate into different cell types, thus repairing/regenerating the damaged tissues [3]. In addition to stemness-associated features, the clinical utility of MSCs is also due to other characteristics, which include (i) the secretion of trophic factors that promote repair of the damaged tissue [4]; (ii) immunosuppressive activity, through secretion of several cytokines that inhibit the activity of natural killer cells, helper T cells, and cytotoxic T cells while activating the generation of regulatory T cells; and (iii) homing abilities, which allow cells to migrate across the endothelium to the sites of injury and inflammation [5].

Due to all these biological properties, MSCs have been widely used in the veterinary field as an Advanced Therapy Medicinal Product (ATMP), demonstrating significant potential in clinical application [6]. Many factors pave the way for their use, including (i) a feasible and relatively easy isolation process; (ii) the lack of immunogenic properties, which permit the usage of allogeneic transplantation in pre-clinical and clinical trials [5]; and (iii) the absence of ethical controversy. However, the costs related to their application, mainly in musculoskeletal and joint diseases, limit their use mainly to the equine and canine species (and, at a lower extent, feline species).

**Figure 1:**
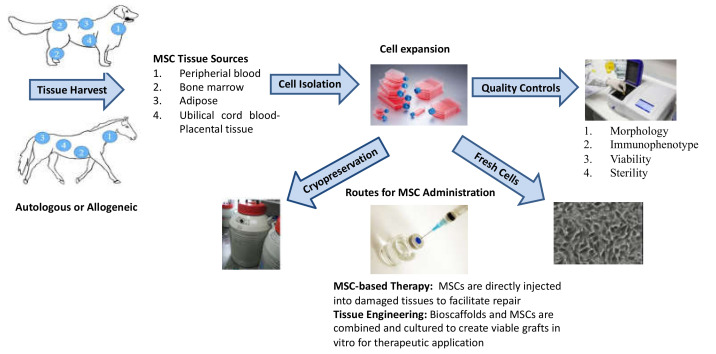
Schematic of equine and canine adult mesenchymal stromal cell (MSC) processing. (Adapted from Duan, W. et al. [8]).

In veterinary medicine, everything regarding tissue sampling, MSC culture, expansion, and cryopreservation is essentially similar to what is commonly applied in human medicine [7,8,9]. A summary panel of the main procedure is shown in Figure 1. Even in this case, many factors can influence MSC therapeutic effectiveness, including tissue source, isolation procedures, culturing conditions (pH, temperature, incubation time, medium supplementary, serum starvation) and cryopreservation. All these must be standardised in order to reduce the variability among the different cell batches.

### 2.1. MSC Isolation Tissue

Experimental evidence indicates that MSCs can be isolated from almost every tissue of the body (comprising bone marrow, adipose tissue, peripheral blood, and extra-embryonic tissues) [10]. The source of MSCs is of extreme importance, as it has been recently reported that MSCs derived from different anatomical sites possess different in vivo differentiation potentials [11] as well as subtle differences in biological features. Nevertheless, clear indications concerning which source is best indicated for a specific disease are not always available, and, for musculoskeletal therapy, it remains controversial which source of MSCs could represent the most valuable and reliable in terms of cell yield and biological features [12].

As in human medicine, bone marrow (BM) has been historically the first and most investigated source of MSCs in horse and dog [13,14]. Yet, a strong disadvantage of BM-MSC is the invasive collection method associated with the risk of complications, such as infection, haemorrhage, and, in the horse, pneumothorax, or pneumopericardium [15,16]. One of the most extensively investigated sources of MSCs is the adipose tissue (AD) [15,17,18,19]. Its success as an MSCs tissue source is due to the easier access, that allows safe and rapid recovery of tissue samples, via lipectomy or lipoaspiration (used for examples in horses and dog) [15,20]. Along with the use AD-MSCs, recently active investigation has been focused on the adipose-tissue-derived stromal vascular fraction (SVF) which is believed to bring some practical advantages over the use of in-vitro expanded AD-MSCs [21]. SVF results easier to isolate and allows to obtain a ready to use the product after minimal contact with xeno-reagents, without the need for any cell separation or expansion, leading to less strict regulatory criteria. One limitation of its application, in substitution of AD-MSCs, is due to the presence of different cell types which can potentially cause immunological rejection so that SVF indeed applicable for autologous treatments only. Nevertheless, in vivo studies have been performed using allogeneic SVF demonstrating its safety and efficacy [22]. In some species, i.e., dog and cat, abdominal visceral fat has been proposed as a source of MSCs. In this case, fat tissue can be collected with a relatively simple surgery from the patient or during elective surgery. As an example, ovariohysterectomies, a common surgical procedure in dogs and cats, has been proposed as a useful approach for the collection of visceral fat [18,23]. Peripheral blood seems to be a valid alternative source compared to bone marrow and adipose tissue for some authors. However, findings regarding the availability of MSCs in peripheral blood are controversial. Positive results have been reported in rabbits, mice and guinea pigs [24], but they stand in contrast to poor success described in humans, dogs, and horses [6,16,25,26]. In detail, one of these studies has demonstrated that fibroblast-like cells isolated from dog, guinea pig, and rat peripheral blood possess a certain capacity to differentiate into several mesenchymal lineages. Equine peripheral-blood-derived fibroblast-like cells, instead, can differentiate into different mesenchymal lineages but have less multipotency than BM-MSCs [26]. Although results need to be studied more extensively, other promising outcomes were obtained from the investigation of the equine synovial membrane and synovial fluid as a potential MSCs source in which cell collection is feasible with a minimally invasive procedure [27]. Finally, knowing that MSCs yield decreases with the increasing age of the donor [28,29], umbilical cord blood (UBC) and matrix, amniotic fluid [30,31], or placental tissue are an auspicious source of MSCs. Equine UCB samples appeared to be a rich source of readily available and highly proliferative MSCs that could be applied for therapeutic use [29]. The disadvantages of these sources are due to their collection procedure, performed in a no-sterile condition environment, at the parturition [32].

### 2.2. MSC Isolation Procedures

As reported above, MSCs can be isolated either from ‘liquid’ sources, such as peripheral blood, or from ‘solid’ sources, such as adipose tissue. In the first case, the isolation process will employ a gradient centrifugation protocol, whereas, for the second one, an enzymatic treatment will be needed. Afterward, cells are seeded in plates for their adhesion, while the non-adhered ones will be wiped out when replacing culture medium with fresh one [7,33].

Depending on the type of cells and technical factors, usually within a week or 10 days, colonies of adherent cells will appear and gradually cover as a layer the culture plate bottom, until they reach the confluence. Cell layers will be then detached by trypsinization and reseeded to permit a continuous proliferation every time confluence is reached; this step is called ‘cell sub-cultivation’, and each one is referred to as passage number. MSCs should not be expanded more than four passages for clinical applications to maintain their stemness; indeed, further passages lead to senescence, meaning lower proliferation and morphological as well as biological changes. For instance, previous studies have shown how the onset of senescence is related to a telomere length reduction; senescence of equine BM-MSCs occurs faster than adipose and umbilical cord-derived MSCs [34]. These results indicate the adipose tissue and, to a lesser extent, for difficulties in the collection, the umbilical cord, as preferable choices for tissue regeneration.

### 2.3. MSC Culturing Conditions

The medium used for MSC culture is mainly represented by the Basal Medium Eagle (BME), Minimum Essential Media (MEM), and Dulbecco’s Modified Eagle’s Medium (DMEM); they are different in the content of amino acids and mineral salts, and the concentration of glucose.

Supplementation of the medium is performed to mimic in vivo conditions in order to sustain cell growth. Foetal bovine serum (FBS) or platelet lysate [35,36] are used in the cell culture medium as a source of growth factors [37], which supports attachment and further expansion of MSCs. The use of antibiotics is important in order to prevent bacterial contaminations, and the most commonly used are penicillin and streptomycin.

The pH value of the medium ranges between 7.2 and 7.4, and in order to keep this value constant, cell plates are usually incubated in 5% CO_2_ atmosphere, and the medium culture contains NaHCO_3_ as a buffer.

### 2.4. Cryopreservation

Cryopreservation is a conservation method which allows storing biological material at very low temperatures (−196 °C using liquid nitrogen or −80 °C using carbon dioxide). Cryopreservation process turns to be useful for the storage of cells for extended periods; indeed, freezing in liquid nitrogen keeps cells alive in a complete quiescence phase for years. By default, it should be considered that cryopreservation alters or compromises the structure and function of cells; in the worst cases, the ice crystals formed during the freezing process can cause cell damage. For this reason, due to the complexity of the whole process (freezing and thawing), a choice of excipients and process parameters, to protect the cell integrity from stresses, is challenging. These excipients are generally named as cryo-protectants and are mainly divided into two categories—membrane-permeable and impermeable. Cryo-protectants with high permeability are, for example, dimethyl sulfoxide (DMSO), ethylene glycol, methanol, propylene glycol, and dimethylacetamide. They tend to be the most cytotoxic. Cryo-protectants less permeable are methylcellulose (MC), polyvinylpyrrolidone (PVP), hydroxyethyl starch (HES), polyethylene glycol, and dextran. In association, FBS is used as a source of protein with synergic cryoprotectant effect [38]. A mixture of DMSO and FBS is considered as the standard cryo-protectant in veterinary medicine, even if the oncogenic and xenogeneic properties of DMSO and FBS, respectively, may alter cells and impact in vitro and in vivo behaviour after implantation [8].

Regarding cell transportation, many studies have shown which are the shipping criteria that should be considered—for instance, supplementing media, transport temperature, and other variables, such as the type of container for the shipping. The latter did not show meaningful evidence; in detail, the use of plastic rather than glass containers did not show differences in terms of cell viability after 24 h at room temperature (20–22 °C) at several MSC concentrations [39]. Shipping temperature is up to cell storage conditions: frozen cells should be transported in dry ice around −80 °C, inside cryovials often wrapped with precooled material and placed in a leak-proof container to prevent direct contact of samples; otherwise, they can be transported in liquid nitrogen to maintain −196 °C temperature. In the literature, it is reported that shipping frozen equine BM-MSCs is the most appropriate method to maintain about 80% of cell viability [40] up to six months [41]. Fresh cells, instead, have been valued differently in several studies: transport was evaluated at different temperature (4 °C, 37 °C, and room temperature 20–22 °C) in PBS or DMEM, either alone [42] or supplemented with FBS or with horse serum [9]. As a result, the majority agreed that MSC transport should be performed at 4 °C, which seems to be the most practical one. According to this, shipping fresh equine MSCs in isotonic saline solution at 4 °C within 24 h is considered ideal for immediate administration, although, in these conditions, MSC viability decreases up to 70% [39,40,43]. Only one study considered room temperature as superior in the case of short-duration transport [43].

The consciousness of differences between the employment of fresh and frozen MSCs is now ongoing. As mentioned before, each step of cryopreservation can affect MSC viability. Concerning this, two reports have demonstrated that while frozen canine AD-MSCs and BM-MSCs had a lower proliferation capacity, lower telomerase activity, and a loss of cells expansion, fresh cells maintained their capacities exploring the same parameters. Similarly, feline cryopreserved AD-MSCs showed lower CD9 and CD105 expression compared to fresh cells [44]. On the other hand, several other studies led to different results. In detail, Martinello et al. proved that, after cryopreservation, equine peripheral blood MSCs conserved their morphology, telomerase activity, proliferation rate, and CD expression pattern [45]. Overall, the use of fresh and cryopreserved MSCs is controversial, and particular care on the cell viability and passage level must be taken before their administration.

### 2.5. Quality Controls

Another important aspect that should be considered when isolating MSCs is their characterization, performing quality, and quantity controls on the starting material and the final product. In such a way, it can ensure the quality, reproducibility, functionality, potency, efficacy, and safety of the clinical product. Required quality controls regard the morphology, immunophenotype, differentiative potential, viability, and sterility testing [46].

### 2.6. Routes for MSC Administration

Although MSCs delivery routes are different, typically, clinical treatment requires the administration of cells using needles. Cells activity and survival could be affected not only by needles type and diameter but, notably, also by following the aspiration and re-injection of MSCs suspension, probably due to the negative pressure during aspiration [47]. Since the passage of MSCs through a needle may affect cell viability, the appropriate catheter aspects should be taken into considerations. Usually, for joint pathologies, for instance, osteoarthritis, MSCs suspension is directly injected intra-articularly. In tendon core lesions, intra-lesional direct administration of MSCs is the preferred route, while administration within the tendon sheath or by regional perfusion with the use of intravenous catheter is used in the case of multi-focal lesions or when the damaged site is difficult to access [48]. Intra-arterial delivery could be effective, but this route is discouraged because of the risk of thrombosis [49]. In the case of focal cartilage lesions, MSCs could be encapsulated into a scaffold and be placed directly into the damage region under arthroscopic guidance [48].

### 2.7. Autologous vs Allogeneic MSCs

There are mainly two approaches for stem cell therapy in regenerative medicine—autologous or allogeneic cell transplantation. An autologous transplant uses the patient’s stem cells, while allogeneic transplant uses stem cells from a donor. Allogeneic versus autologous MSCs is a hot topic in veterinary regenerative medicine, debating whether there is a difference in terms of safety and efficacy. The use of allogeneic MSCs is subjected to specific national regulations, which are important to consider for trials and clinical applications. However, the use of allogeneic MSCs would represent an important advantage offering availability of banked cells, previously characterised for their safety and biological features, as a proven differentiation capacity. This approach would reduce the typical variability of autologous cellular products, allowing greater homogeneity in treatments and, presumably, results. Moreover, by this approach, it is possible to have a ready-to-use product for appropriate timing of therapeutic applications, without the need to wait for the autologous cell culture expansion.

Using the patient’s cells to treat an injury or disease is believed to be safer, but on the other hand, one of the major challenges of this approach is due to timing. Indeed, the timing to expand the right amount of cells for implantation is highly dependent on the harvested tissue, as well as on their reproduction capacity, and thus by age, gender and disease type [50].

Some trials have been conducted on different animal models to compare allogeneic and autologous MSCs. In one of them, self and non-self placenta-derived MSCs were injected into contralateral joints of 16 healthy horses. A comparison was made in terms of lameness evaluation and synovial fluid analysis from 0 h up to 72 h post-injection. The injection of allogeneic MSCs did not provoke a systemic response, while local response such as swelling was minimal, and inflammatory response was not significantly different between the two treatments. Thus, this pre-clinical work is an important step in the development of equine allogeneic stem cell therapies [51]. Furthermore, Shah et al. reported the outcome of the treatment conducted on 203 dogs suffering from osteoarthritis and other joint defects with allogeneic stem cells derived from adipose tissue. Dogs of various breeds and different ages were enrolled in the study. Most of the patients received an intra-articular therapy, while 68 patients were treated intravenously. The large majority of younger animals (90% of < nine-year-old dog) and a large percentage of older patients treated with the allogeneic adult stem cells improved symptoms and demonstrated better quality of life. Only a single patient had a worsening of the symptoms. The large number of dogs enrolled in the study, and the administration routes of the cells, point out the safety of the allogeneic treatment, although the follow-up was limited to 10 weeks [52]. The result of this report strengthens similar findings, where allogeneic MSCs have been able to aid the body’s regeneration abilities without causing an immune response or other adverse effects [52,53]. Another proof of allogeneic MSCs safety has been reported by Brandão et al., who analysed autologous and allogeneic AD-MSCs in order to evaluate the inflammatory response in healthy equine tendon [54]. The outcome in the two groups has been compared to a control group where only PBS was administered. After injection, all the groups presented mild pain sensitivity on the second day, which can be explained as a consequence of the healthy tissue response to an injury application. There were no significant differences among the groups in the physical, morphological, thermography, and ultrasonography analyses. Moreover, even the lameness analysis presented similar behaviour between the two cell-treated groups. Interestingly, the authors analysed tissue and cellular response to MSCs administration, concluding that both allogeneic and autologous AD-MSCs did not induce a significant inflammatory response, although a higher number of T lymphocytes have been observed in the group treated with allogeneic cells. Based on their result, the authors suggest that allogeneic MSCs did not have adverse effects in comparison to the autologous cells, thus reinforcing the hypotheses that allogeneic banked cells could be a safe and effective approach to regenerative therapies. According to this, a similar conclusion came from a different study conducted on allogeneic BM-MSCs transplantation in equine tendons. The analysis showed no significant differences compared to the autologous cells [53]. In this latter study, small lesions were created in the equine superficial digital flexor tendon, followed by injection with autologous, allogeneic, or bone marrow supernatant alone, respectively. Post-mortem examinations revealed there was no cell-mediated immune response to the host for both autologous and allogeneic MSC treatment.

These results seem to be promising and lead to the conclusion that allogeneic rather than autologous MSCs could be used for regenerative medicine purposes in veterinary medicine. Hence, it would be important to implement studies related to the immunomodulation of MSCs in order to understand cell response better and reinforce, accordingly, therapeutic applications. In particular, immunoreactivity tests performed both in vitro and in vivo using allogeneic MSCs are important to guarantee safe and effective therapy. Moreover, further in-depth studies still need to be performed to understand the real behaviour of the stem cells at the site of application and their crosstalk with resident cells [55].

Finally, most of the clinical applications to investigate MSCs behaviour are conducted on experimentally damaged tissue models. Some authors, indeed, suggest that to evaluate MSCs action mechanisms and attitude better, it would be necessary to apply cells in healthy tissues [53]. The injection of allogeneic MSCs in the considered area would allow the assessment of the possible local inflammatory reaction. Thus, this could reveal the interaction of any other aspects, proving that alterations are caused by cell transplantation [56]. Working on healthy tissues is also important to predict the side effects of allogeneic MSC transplantation in animals. All the considered studies regarding autologous vs allogeneic MSCs are summarised in Table 1.

## 3. MSCs in the Veterinary Field: Disease Targets

Musculoskeletal disorders (MSDs) represent common pathologies in veterinary clinical practice. MSDs affect the osteoarticular apparatus, including muscles, bones, and joints. They are associated with painful symptoms that can be both acute and chronic. These disorders often result from overuse injuries, muscle fatigue, inflammation of the tendon structure, or intervertebral disk degeneration of the vertebral column. Examples of MSDs include osteoarthritis (OA), tendon ligament injury (TLI), and intervertebral disk degeneration (IVDD) [57].

Up to now, treatment options for MSDs include systemic or intra-articular administration of anti-inflammatory drugs, hyaluronic acid (HA), cells-based products including platelet-rich plasma (PRP), and autologous/ allogeneic cells implantation [57,58]. Since MSDs have a high prevalence, regenerative therapies, including the use of MSCs, have been brought to the attention of veterinary practitioners as an alternative to the more traditional treatments [54,59,60]. Below, the employment of MSCs to treat each specific MSD is reported.

### 3.1. Osteoarthritis

OA is the most common form of arthritis related to the progressive degeneration of articular cartilage and subchondral bone, leading to severely debilitating conditions. It is a chronic and irreversible condition involving the cycle of inflammation and tissue degradation [61].

In recent years, MSCs have been proposed as a therapy for the treatment of OA in both dogs and horses. The currently available evidence of the MSCs effectiveness and safety profile is confirmed in the clinical trials reported in Table 2. In all cases, whether it is a dog or a horse, veterinarian practitioners directly administered MSCs into the joint by intra-articular injection [40,62].

Regarding dogs’ treatments, the most common approach is to use adipose tissue as cells source, favoured by the possibility of minimal-invasive collection. In detail, as reported in a review, different clinical trials on the use of AD-MSCs have been investigated comparing the AD-MSCs alone or in combination either with intra-articular autologous PRP or HA as a chondroprotective agent [63]. Considering the common outcomes of the studies reported in Table 2, the most important element is the safety and efficacy of the therapy, expressed in term of the absence of any side effects (including local and systemic inflammation), reduction of lameness, improvement of joint functionality, and pain reduction. As a general trend, better endpoints were noticed in dogs treated with AD-MSCs associated with plasma rich in growth factor (PRGF-Endoret) [64] than AD-MSC alone [65]. It has been indeed demonstrated that in vitro PRP releases growth factors, including Transforming growth factor beta (TGF-β), platelet-derived growth factor (PDGF), epidermal (EGF), and insulin-like growth factor (IGF) that affect cartilage regeneration [66]. Another study demonstrated the superiority of AD-MSCs alone over PRP at the six-month follow-up, although outcomes were not described beyond this period [67]. OA in dogs has also been treated by injection of AD-MSC suspended in saline solution, comparing the outcome to dogs treated using saline solution only, as a control group. This experiment was reproduced afterwards by the same group of research administering a higher amount of cells and with a longer follow-up. In fact, since the pain grade of the affected joint was severe, the success rate after the injection was not significantly high in the first study; however, clear evidence of the efficacy of MSCs therapy was observed in the second work [68,69].

To examine the impact of MSCs in the treatment of equine model of OA, several clinical studies have been performed. A recent review compares intra-articular injection of MSCs in both naturally occurring and induced equine OA; results have been variable, which may be caused by the changing environment, follow-up, MSC dosage and source, as well as inter-observer differences in subjective outcome parameters [70]. Considering bone marrow as MSCs source, autologous cells have been dispersed in HA, and their effect has been compared to the HA alone used as a control [71]. Horses had defects arthroscopically created on both stifle joints and received an intra-articular injection; after 12 months, horses were euthanised. Results showed no significant improvement, but evaluation post-mortem performing histologic and immunohistochemical analyses confirmed a significant increase in joint repair. Naturally occurring OA treated with BM-MSCs dissolved in HA have also been investigated, post-surgery (arthroscopy), supporting the amelioration of horse condition after cells injection even after 24 months [72]. When treating naturally occurring OA, there is more variation because duration and severity of the disease vary, and it is difficult to judge whether or not the treatment with MSCs results efficacious and in what extent [73]. Such conditions often lead to a lack of objective conclusions due to, for example, of variation in joints treated and lack of control groups [41]. Comparison between cell treatments and elective drugs has been explored; in detail, the injection of autologous AD-MSC versus steroid drugs (betamethasone) was evaluated up to 180 days [74]. At the end of the period, no inflammatory response was observed in any groups, and improvements were noticeable in AD-MSC treated horses but not in the one cured with betamethasone and group control. Recently, Broeckx et al. [75] proposed a somehow alternative approach to the therapy of equine degenerative joint disease. Their randomised, double-blinded, and placebo-controlled clinical trial enrolled 75 horses affected by fetlock joint osteoarthritis. The treated group of fifty horses received a single intraarticular injection of blood-derived, allogeneic chondrogenic-induced MSCs. Cells were resuspended in allogeneic plasma. The authors report better scores for treated animals regarding lameness, flexion test, joint effusion both at short (3–18 weeks) and long-term (one year) follow up. The novelty of this study lies in the use of blood-derived MSCs, to replace the most widely used adipose-tissue or bone marrow-derived cells and their combination to allogeneic plasma to improve cell viability, replication, and chondrogenic differentiation. All the MSCs were prepared from a single donor and pre-differentiated at P9; no adverse effects are reported following the application of allogeneic MSCs and plasma.

### 3.2. Tendon Ligament Injury

Tendon ligament injury (TLI) affects a large part of the equine population ranging from acute traumatic ruptures to chronic overuse and degenerative tendinopathies [76]. The outcomes of conventional therapies are quite often unsatisfactory due to the poor regeneration capacities of tendon tissue in equine species [77,78]. In fact, apart from a primary inflammatory reaction following the injury, spontaneous healing is characterised by fibroplasia and can be referred to as a repair rather than a regeneration process [79]. The repairing system leads to the formation of cellular scar tissue with low extracellular matrix organization, in which stiffness is increased, but elasticity is decreased compared to the original tendon tissue [55,79,80,81]. Indeed, the primary outcomes investigated to evaluate tendon regeneration are the stiffness, modulus of elasticity, histological score, DNA content, vascularity, and compositional parameters, which could be considered indicators of regeneration, when compared to levels observed in the normal tendon.

In recent years, research has focused on regenerative therapies and tissue engineering approaches, with the aim to recover the original function of the damaged tendon. Typically, as the tendon core lesion is clearly visible by ultrasonography, the application of MSCs is simply performed by injection of the cell suspension directly into the damaged tissue [82]. Specifically, some studies have been conducted to evaluate the role of MSCs on equine tendon healing, injecting cells derived from different sources (mainly adipose tissue and bone marrow), autologous or allogeneic, alone or associated with other treatments (PRP, HA) [80,83]. Although most of the reports are not blinded or do not provide sufficient information about controls, the literature describes encouraging outcomes, giving evidence of the benefit and safety of MSC application for tendon regeneration [84] MSCs treatment has been described either in healthy animals, in which experimental lesions were induced surgically or by collagenase gel in the superficial digital flexor tendon [80,85]; or in naturally occurring TLI [84,86,87]. Romero et al. evaluated tissue healing in an experimental tendonitis model after administration of autologous bone marrow and adipose tissue-derived MSCs and platelet-rich plasma (PRP). BM-MSCs and PRP produced similar results, although PRP-treatment resulted in higher expression of COL3A1 and ACAN genes, suggesting lower tendon regeneration. Although all the treatments showed beneficial effects compared to the control group, the authors concluded that BM-MSCs might provide better healing properties [88]. Brandao et al. [45] studied the local inflammatory response of tendon injected with autologous or allogeneic AD-MSCs, concluding that no adverse or inflammatory reaction was observed in horses treated with allogeneic cells. Actually, allogeneic cells have been extensively used also for the treatment of naturally occurring tendon lesions supporting the safety of allogeneic cells administration in the horse [85,86,87,89]. In most clinical studies, outcomes are evaluated comparing results to conventional therapies in term of re-injury rate following the return to activity. Although this approach does not give an accurate indication about the quality of tissue recovery, it provides practical information about the possibility of the animal to return to their previous normal activities. Interestingly, cell treatment resulted in a significantly lower re-injury rate in comparison to conventional therapies. Generally, re-injury rate following traditional treatment such as hyaluronan, beta aminopropionitrile fumarate or polysulfated glycosaminoglycans, ranges between 23–80% [77,90], while the re-injury rate following MSCs medication is reported to be lower. In this regard, Pacini et al. observed that nine out of 11 racehorses could return to competition without any re-injury event, within a follow-up period of two years [91]. Similar results have been observed in a different clinical report, where after MSCs treatment, the majority of the patients returned to their previous activities, avoiding re-injury [84]. Smith et al. conducted a clinical study on 82 racehorses and 24 other sports horses; after rehabilitation follow-up, only 13–36% of the horses were re-injured, depending on their disciplines [92]. Smith et al. [81] compared the mechanical and morphological characteristics of the tendon extracellular matrix in horses affected by spontaneous tendonitis. BM-MSC treated tendons demonstrated improvements in several parameters compared to not treated tendons, providing evidence on the role of cell therapy on tendon healing in naturally occurring tendonitis.

Regarding tendinopathy in canine clinical reports, the scenario is way less documented and investigated than horses; nevertheless, the beneficial effects of MSCs seen in few studies, are quite significant. Although tendonitis has not a high prevalence in dogs, supraspinatus tendinopathy (ST) represents a quite common cause of forelimb lameness. Aetiology is probably related to overuse from chronic repetitive activity. Canapp et al. applied adipose-tissue-derived MSCs in combination with PRP to the treatment of 55 dogs with ST [93]. Based on ultrasonography and objective gait analysis results, the authors suggest that MSCs administration is a promising therapy for ST. More recently, the same group extended the study to the use bone marrow aspirate concentrate (BMAC) combined with PRP, observing positive sonographic results with improvement of tendon size (significant reduction of the affected tendon), fibre pattern and echogenicity, even if only 13.8% dogs treated recovered entirely to a normal fibre pattern at 90 days post-treatment [94]. This could be related to the short follow-up in comparison to the longer time needed for tendon healing.

Finally, a study made a comparison between BMAC and AD-MSC added to PRP for the therapy of cranial cruciate ligament tear in dogs [94]. In this research, the dogs recruited were different in terms of breeds, age, weight, and sex; the outcomes were based on diagnostic stifle arthroscopy, radiographs, orthopaedic examinations, gait evaluation, and functional questionnaire. Results proved cells efficacy, but there are limitations due to a lack of standardization of PRP preparations the uncertain number of administered cells. This could have affected the outcomes of the two treatments (AD-MSC and BM-MSC). While all these results are encouraging, the long-term success of MSC treatment remains to be proven.

Although semitendinosus myopathy is not tendonitis, this fibrotic musculoskeletal disorder of working dogs affects a muscle whose long tendon is part of the Achilles tendon. Gibson et al. recently conducted a study using a single administration of AD-MSCs to treat semitendinosus myopathy in 11 police dogs, comparing follow-up at six months and one year. At six months follow-up, all patients had returned to work, while at long term follow-up, the dogs were still active in their previous activity, showing an improvement in gait [95]. The authors do not report a recurrence of the disease, and most of the dogs worked until retirement, suggesting a long-term efficacy of the therapy. Table 3 reports all the considered studies regarding TLI treated with MSCs.

### 3.3. Intervertebral Disk Degeneration

Intervertebral disk degeneration (IVDD) is a complex multifactorial process considered as the primary cause of lower back pain in humans. Since intervertebral disk degeneration is incurable, all available treatment strategies are mainly focused on pain relief only [97]. Therefore, efforts are dedicated to the development of strategies able to regenerate, or at least repair, and preserve the functioning of the intervertebral disk structure. Among these, intradiscal injection of MSCs has become highly topical in experimental and clinical investigations.

Dogs with IVDD are the only patients where medical and surgical approaches similar to humans are used [63]. For this reason, canine IVDD is considered a valuable and reliable disease model for the investigations of novel and effective healing treatment for human IVDD. In a recent study by Steffen et al. [98], six dogs suffering from naturally occurring degenerative disc disease received autologous bone-marrow-derived MSCs. Although results showed no adverse effects, the authors were not able to demonstrate by MRI any apparent regenerative effect of the treatment. This work was pioneering in naturally occurring IVDD, thus negative result may be caused by biological and biomechanical differences between injury-induced and naturally occurring IVD degeneration. To improve the therapeutic approach, the authors, in a following study [99], introduced collagen microcarriers as a scaffold for MSCs. Twenty dogs affected by spontaneous lumbosacral IVD degeneration confirmed by MRI and clinical signs (lumbosacral back pain) were included in the study. Autologous MSCs were isolated from bone marrow and, before the injection, MSCs were mixed with collagen microcarriers, as a delivery system, with or without TGF-β1 crosslinking. After decompressing surgery, dogs were divided into three groups, which received three different intradiscal injections of 1) intradiscal injection of MSC-microcarriers, 2) MSC-TGF-β1-microcarriers, of 3) microcarriers only. Clinical performance and Pfirrmann grading, assessed through magnetic resonance imaging, were evaluated at 10 months after the injection. In vivo injection was successful in all dogs, and clinical functioning returned to normality. However, post-operative Pfirrmann grade remained unchanged in all dogs, and the undesired side-effect formation of Schmorl’s nodes occurred in 45% of the dogs. This side effect was reduced by halving the injection volume. Therefore, clinical improvement was observed in all groups, despite the formation of Schmorl’s nodes, but microcarriers and MSCs failed to regenerate the structure of degenerated IVD [99]. A successful work was conducted using fetal allogeneic BM-MSCs on seven dogs in which, by the end of the period of 90 days, all dogs had an improvement in functional movements and were able to take steps; moreover, none of them presented adverse symptoms at any time [100]. Two years later, a randomised control case report was conducted on 34 dogs suffering from IVDD disorder with no deep pain; in this study, surgery was evaluated alone and in combination with transplantation of allogenic AD-MSC into the spinal cord. Neurological progress was noticed, and the success rate for the AD-MSCs group was significantly higher (77.8%) than surgery alone, thus demonstrating the potential therapeutic efficacy of MSCs [101].

Even though the use of MSCs in veterinary regenerative medicine for IVDD treatment seems to be promising, as mentioned above, there are also clinical results that suggest that MSCs are not always capable of repairing the damaged tissue, at least using the therapeutic protocols proposed so far [102]. Table 4 reports all the considered studies regarding IVDD treated with MSCs.

## 4. Animal Spontaneous Pathologies as Potential Preclinical Models for Human Therapy

Experimental animal models are still widely used to study biological properties and therapeutic potential of MSCs in regenerative medicine. From this point of view, a key point is the choice of the animal species to develop a useful and scientifically relevant model. Resorting to laboratory animal species (mice, rats, rabbits) to establish the safety and efficacy of novel therapeutic strategies leads to the use of induced experimental models very far from the clinical reality of human medicine, making their use uninformative and leading to the sacrifice of a high number of animal lives. This aspect is in contrast with the European Parliament directive (Directive 2010/63/EU) which limits the use of animal models and suggests to the scientific community the use of alternative methods, as a result of the 3Rs principle promoted by Russell and Burch in 1959 [103].

Using spontaneous animal pathologies may be a strategy. Specifically, referring to naturally occurring, spontaneous diseases of domestic animals (primarily dog, cat and horse) allows using model diseases much more similar to human ones from pathogenesis, evolution and biological mechanisms of healing. Thus, companion animal clinical models represent an important contribution to human studies [104]; indeed, similarities concerning symptoms, etiopathology, biomarkers [105] and even genetic [63] can be found with the human counterpart. Also, dogs and cats share with humans the living environment and underlying pathologies (e.g., obesity, diabetes) which often influence the pathological onset, evolution, and healing processes. Finally, their relatively long life expectancies make companion animals a model for long-term studies. Using spontaneous pathologies of these animals for the development of innovative regenerative therapies is, therefore, of extreme interest for human and veterinary medicine, thus avoiding experimental animal models.

Musculoskeletal disorders are an important example of this concept. Cell therapies based on the application of MSCs aim to regenerate damaged tissues exploiting tissue’s intrinsic potential for repair. The complexity of the healing processes induced by MSCs makes it necessary to use natural pathologies (and not experimentally induced disease models) to understand the real therapeutic potential of the cells. In dogs and horses, musculoskeletal disorders have a high prevalence and are considered quite similar to those developed in humans. Innovative cell therapies for osteoarthritis, tendonitis, and intervertebral disk diseases are actively investigated in veterinary medicine, providing useful therapeutic protocols for human medicine [106]. A further advantage of the use of spontaneous diseases to assess the efficacy of MSCs treatment is not only that experimental animals are preserved but affected companion animals enrolled in the studies can receive up-to-date therapeutic opportunity and long-term follow-up.

## 5. Secretome and Extracellular Vesicles as a Potential Therapy for Different Disease Areas

During the last decade, it has been demonstrated that MSCs therapeutic effectiveness is mainly due to the release of paracrine factors, named secretome, composed of free soluble factors (including cytokines, chemokines, and growth factors) and non-soluble nano/microstructured extracellular vesicles (EVs) [107,108]. Several studies support the hypothesis that the EVs fraction alone may be sufficient to heal the injured tissue or prevent tissue damage in several contexts [49]. EVs isolated from MSCs exhibit the same functions as stem cells, for example, their anti-inflammatory and pro-regenerative activity [2,109,110]; but they also can elide some issues related to the usage of the whole stem cell. In detail, they have lower immunogenicity, smaller size, and so they could represent a safer alternative compared to the cell injection [111,112,113]. Despite these advantages, more studies need to be conducted to elucidate kinetics, mechanisms of action, bioavailability (including factors like dosage and frequency), and route of administration. EVs delivery could be provided by using suitable materials and methods such as the encapsulation within hydrogel or scaffold, improving EVs immobilization plus frequency and dosage of administration.

However, this approach, especially in the veterinary sphere, is still in its early stages. Only recently, the therapeutic use of the MSCs-secretome, instead of the parental cells, has been proposed for veterinary applications, also overcoming the practical difficulties related to stem cell application. In detail, based on the hypothesis that cells may promote tendon repair via paracrine factors, an interesting study has been recently published regarding the secretome profile in eight dogs [114]. In detail, exosomes and soluble factor derived from BM-MSCs and AD-MSCs of the same canine donor have been described and compared for the first time, paying attention also to their immunomodulatory capacity. Both cells types share analogy as morphology, but they also have their biological features such as gene expression and proliferation and differentiation. Outcomes showed a higher proliferation rate for AD-MSCs, whereas the production of exosomes and soluble factors, comprising several cytokines, was more active in BM-MSCs. These results were also confirmed by the proteomic analysis. The limitation of this work was the small number and size of the animal model; nevertheless, it is the first step toward secretome characterisation and application [115]. In this contest, El-Tookhy et al. investigated the exosome and microvesicles role in wounds healing process of experimentally induced critical size defects in six dogs. Wound reduction size was observed in 14 days comparing skin area treated with exosomes and the one treated with PBS as a control group. As expected, photographs and histopathological evaluation showed better and faster endpoints of the healing process in dogs treated with exosomes, in particular about the formation of extracellular matrix, angiogenesis, and re-epithelization. The findings underline the efficacy and safety of cell-free therapy in wound repair, paving the way for future new therapy approach that overcome the limitations associated with the use of cells implantation [116].

As far as we know, Lange-Consiglio et al. were the first group to test in vivo the immunomodulatory features of conditioned medium from amniotic membrane-derived MSCs (AMC-CM) evaluating the beneficial effects in thirteen horses affected by spontaneous tendon or ligament injuries [117,118]. All horses included in the study had lesions graded from four to six, presenting an advanced injury stage. Intralesional treatment was well-tolerated, no adverse reactions to the AMC-CM injections occurred, and no soreness was noticed, thus confirming that the treatment was well tolerated in the short-term period. Moreover, the absence of abnormal fibrotic and metaplastic tissue at any time post-injection in the treated area underlines the safety of the secretome administration. Treatment outcome was successful for all the patients considered; in detail, six out of thirteen horses were healed entirely and resumed their previous activity within 4–5 months post-treatment. This latter study suggests a new approach of cell-free treatment for tendon and ligament diseases, obtaining advantages with respect to cell therapy. Apart from a higher safety profile of their administration and their lower immunogenicity, such EVs benefits occur because of the possibility of an easy production of large quantities, and a high storage efficacy, thanks to the possibility to prepare freeze-dried secretome [2,109,119]. Indeed, the lyophilization process allows obtaining a ready-to-use product, causing minimal damage to the substances sensitive to the high temperature and increasing the shelf life of the products.

**Figure 2:**
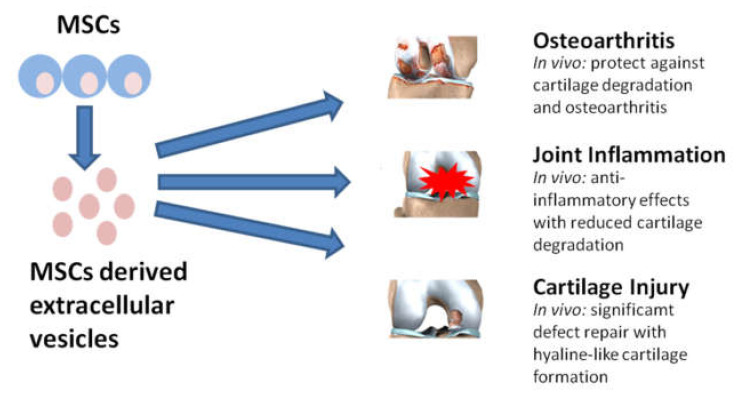
Regarding the human field, stem-cell-derived extracellular vesicles (EVs) can exert a multitude of beneficial effects in the treatment of osteoarthritis, joint inflammation, and cartilage or osteochondral injury. (Modified from Phinney, D.G. [120]).

All the previous considerations lead to a statement: secretome is taking on the role of a new potential therapeutic strategy in different diseases [120,121]. Avoiding the necessity of living cell implantation, secretome shows promising outlook to be prepared as pharmaceutical products suitable for regenerative medicine [122,123]. In particular, EVs are considered a new therapeutic tool having a prominent role in joint and musculoskeletal disorders (Figure 2). Besides all these considerations, up to now, in veterinary medicine, the clinical use of MSC-EVs, although very promising, is still in its infancy, despite numerous pre-clinical models have been proposed.

A key aspect which is missing for EVs and/or secretome pharmaceuticalization is a standardised, reproducible and GMP-compliant production process, in order to obtain validate products suitable for future clinical trials. Isolation method and characterization process are reported in human literature, but unfortunately, in the veterinary field, more evidence is required to confirm the feasibility of this innovative cell-free treatment [2].

## 6. Conclusions

MSCs belong to body repair system and are defined as multipotent progenitor cells able to self-replicate and to differentiate into different cell types, demonstrating significant potential in clinical use. In the veterinary field, many studies have been conducted for the development of the most effective procedures for MSC-based treatment, taking into consideration cell tissue sources, isolation process, culturing conditions, cryopreservation, cell dosage, administration route, and frequency. As a matter of fact, in the last decade, MSCs have emerged as a promising therapeutic tool for the treatment of musculoskeletal pathologies in veterinary medicine. Interestingly, the results accumulated so far have provided evidence that veterinary patients affected by naturally occurring diseases should provide more reliable outcomes of cell therapy than laboratory animals, thus allowing translating potential therapies to the human field. More recently, a cell-free therapy based on MSC- secretome has been proposed. Even though there are very few clinical reports to refer to in veterinary medicine, recent acquisitions suggest that MSC-derived products may have major advantages compared to the related cells, e.g., they are considered safer and less immunogenic. Although several studies propose secretome as a novel therapeutic biological product, a better understanding of the nature, bioavailability, and the mechanisms of action responsible for the beneficial effects of the paracrine molecules is needed. Based on these sceneries, a secretome GMP-compliant production process is needed for veterinary clinical use and further studies on in vivo animal models; indeed, a veterinary medicinal product based on stable secretome formulation represents a valid strategy for MSDs therapy.

## Figures and Tables

**Table 1:** Summary of autologous vs allogeneic MSCs.

Study	Animal	Study Design	Time	Outcome	Dosage
Carrade et al. [51]	Horses	Autologous vs Allogeneic placenta-derived MSCs	0–72 h post-injection	Allogeneic MSCs did not provoke a systemic response, and the minimal inflammatory reaction was found to be similar to the autologous effect	7.5 × 10^6^ in 2 mL sterile injectable 0.9% NaCl
Shah et al. [52]	Dogs	Allogeneic adipose tissue (AD)-MSC	10 weeks	Better quality of life also demonstrating the safety of the allogeneic treatment	Data not recorded
Guest et al. [53]	Horses	Autologous and Allogeneic progenitor cells (MPCs) purified from bone marrow And bone marrow supernatant alone	10 or 34 days	Post-mortem examinations showed no visible cell-mediated immune response to allogeneic MPCs in any of the treated horses	1 × 10^6^ cells suspended in 0.5 mL of autologous bone marrow supernatant
Brandão et al. [54]	Horses	Autologous and allogeneic AD-MSCs and only PBS as a control group	6 days	All groups presented mild pain sensitivity, there were no significant differences among the groups in the physical, morphological, thermography, and ultrasonography analyses. Also, the lameness analysis presented similar behaviour between the two cell-treated groups. Both allogeneic and autologous AD-MSCs did not induce a significant inflammatory response, although a higher number of T lymphocytes have been found in the allogeneic treatment.	1 × 10^7^ cells for each application resuspended in PBS.

**Table 2:** Summary of osteoarthritis (OA) disease treated with MSCs.

Disease	Animal	Treatment	Route and Dosage	Outcome	Ref.
AO- hip	Dog (n = 8)	Autologous AD-MSCs in combination with plasma rich in growth factor (PRGF-Endoret)	Intra-articular injection of over 30 × 10^6^ AD-MSC	Reduced of lameness and absence of side effects for all the period (six months).	[64]
AO- hip	Dogs (n = 15)	Autologous AD-MSCs alone	Intra-articular injection of over 30 × 10^6^ AD-MSC	Reduced of lameness only in the first month (less than three months).	[65]
AO- hip	Dogs (n = 39)	Comparison between AD-MSCs versus PRGF	Intra-articular injection of 30 × 10^6^ AD-MSC	Dog’s pain was reduced, physical function was improved, and no side effects were found. AD-MSC showed better results in the period considered (six months).	[67]
AO- hip (coxofemoral joints)	Dogs (n = 4)	Autologous AD-MSCs in phosphate-buffered saline (PBS)	Dogs received intra-articular injection of either suspension of 4.2–5 × 10^6^ (depending on cell yield) AD-MSCs in 0.6 mL PBSor only 0.6 mL of PBS as a control group	Significant improvement in lameness compared to the control group in the considered period (three months).	[68]
AO- humeroradial (elbow) joints	Dogs (n = 14)	Autologous AD-MSCs in phosphate-buffered saline (PBS)	Dogs received an intra-articular injection of 3–5 × 10^6^ (depending on cell yield) AD-MSC in 0.6 mL PBS	Significant improvement in lameness, range of motion, and pain on manipulation over time (six months).	[69]
OA-stifle injury (femoral condyles)	Horses (n = 10)	Autologous bone marrow (BM)-MSCs in hyaluronan (HA)	Intra-articular injection of either 20 × 10^6^ BMSCs with 22 mg of HA or 22 mg of HA alone	In the period of 12 months: no evidence of clinically significant improvement but arthroscopic evaluation confirmed a significant increase in tissue repair. Immunohistochemical analysis demonstrated more aggrecan levels in the repaired tissue treated with BM-MSC.	[71]
OA	Horses (n = 16)	Comparison between autologous AD-MSC versus steroid drugs (Betamethasone)	Intra-articular injection of 3 groups: (1): 1 mL of AD-MSC in normal saline, at a concentration of 5 × 10^6^ cells/mL (2): 1 mL of betamethasone (3): control untreated	No change in lameness at 30 days but reduced at 60 days. At the period of 180 days, improvement remained in AD-MSC group but not in the steroid group. In the control group, the level of lameness did not change.	[74]
OA- degenerated stifle, fetlock, pastern and coffin joints	Horses (n = 165) In detail: stifle (n = 30), fetlock (n = 58), pastern (n = 34) and coffin (n = 43) joints	Allogenic peripheral blood MSCs with or without chondrogenic induction in combination with PRP	Intra-articular injection. Dosage not stated	Considering 180 weeks period: no adverse effects were noticed, except for three patients. Already after six weeks, 45% (native MSCs) and 60% (chondrogenic-induced MSCs) of the treated patients returned to normality, and the beneficial effects further increased after 18 weeks (78% for native MSCs and 86% for chondrogenic induced MSCs).	[41]
OA-stifle injury (femorotibial lesions (meniscal, cartilage or ligamentous)	Horses (n = 33)	Autologous BM-MSCs post-surgery (arthroscopy)	Intra-articular injection of 15–20 × 10^6^ BM-MSC in autologous serum/5% DMSO + HA compared to surgery alone	Considering 24 months of follow up: Improvements in ability were realised with BMSC treatment compared to surgery alone.	[72]
OA- degenerative fetlock joint disease	Horses (n = 75)	Allogeneic chondrogenic induced MSCs added to allogeneic plasma (EAP)	Intra-articular injection of 2 × 10^6^ allogeneic chondrogenic induced MSCs with EAP	After long-term follow-up (one year), horses were returned to their previous level of work.	[75]

**Table 3:** Summary of tendon ligament injury (TLI) disease treated with MSCs.

Disease	Animal	Treatment	Route and Dosage	Outcome	Ref.
TLI- superficial digital flexor tendon (SDFT)	Horses (n = 8)	AD-MSC suspended in platelet concentrate (PC)	Intralesional administration of 10 × 10^6^ AD-MSC in in 1 mL of PC. 1 mL of PBS was used as a control group	After 16 weeks improvements were reported for AD-MSC group. In detail: decrease of the lesion progression and inflammatory reaction, better organization of collagen fibres, an increase of blood flow. No difference in terms of gene expression was found.	[80]
TLI- SDFT	Horses (n = 141, racehorses)	BM-MSC resuspended in their bone marrow supernatant	Intralesional BM-MSC injection was performed resuspending cells in their bone marrow supernatant at the concentration of 5 × 10^6^ cells/mL.	Two years follow up: no side effects; the need for reinjury was lower than other published works.	[84]
TLI	Horses (n = 6)	Allogeneic AD-MSCs	Injection of 100 × 10^6^ allogeneic AD-MSCs via atlanto-occipital (AO) and lumbosacral (LS) injection	AD-MSCs administration was safe. No alterations in blood and neurological examinations at any time (30 days) either with AO or LS injections. OA had better distribution.	[86]
TLI	Horses (n = 10)	Allogeneic AD-MSC compared with BM-MSC	Intravenous injections of three doses of 25 × 10^6^ allogeneic AD-MSC and BM-MSC respectively	After the first injection, horses were followed up for 35 days. Evaluation was made on the inflammatory and immune response showing that repeated BM-MSC injection increased blood CD8+ T-cell numbers.	[85]
TLI- SDFT (forelimbs)	Horses (n = 12)	Comparison between autologous AD-MSC, BM-MSC, and platelet-rich plasma (PRP)	Injury injections of 20 × 10^6^ BM-MSCs or AD-MSCs suspended in 7 mL of lactated Ringer’s solution (LRS), and 7 mL of PRP. 7mL of LRS was used as control	After 45 weeks, all treatments had beneficial effects, but in detail, data suggest BM-MSCs might be the better approach for tendon healing.	[88]
TLI- suspensory ligament (SL)or superficial digital flexor tendon (SDFT) lesion	Horses (SL *n* = 68) (SDFT *n* = 36)	Tenogenically induced allogeneic peripheral blood (PB)MSCs combined with PRP	intralesional injection of 1ml of PB-derived MSCs (containing 2-–3 × 10^6^) with 1 mL of PRP	In two years, no adverse effects have been observed. At 12 weeks, results were convincing in lesions improvement where about 80% of both SL and SDFT groups went back to their previous performance.	[87]
TLI-SDFT	Horses (n = 11)	Autologous BM-MSC	Injections of at least 1 × 10^6^ of BM-MSCs were re-suspended in 1.5 mL of autologous serum	Patients were back to their sports activities, without having suffered a re-injury	[91]
TLI- SDFT	Horses (n = 12)	Autologous BM-MSC suspended in 2 mL of BM supernatant	Implantation of 10 × 10^6^ BM-MSCs were suspended in 2 mL of citrated BM supernatant. 2 mL of PBS were used as a control group	In six months, there were significant benefits in terms of safety and healing tendon process (reduced stiffness, histological showed better organization and reduction in re-injury rate).	[81]
TLI- supraspinatus tendinopathy	Dogs (n = 41)	BM-MSCs in combination with PRP	Ultrasound-guided intratendineous injection of BM-MSCs with PRP (1:1 ratio)	On 90 days post-treatment: in most cases, the fibre pattern and echogenicity have improved, while only a minority resolved fibre pattern and echogenicity abnormalities.	[96]
TLI- partial cranial cruciate ligament tear	Dogs (n = 36) 19 cases received BM-MSC while 17 cases received AD-MSC	Autologous BM-MSCs vs AD-MSCs were combined with platelet-rich plasma (PRP) in 1:1 ratio when injected.	2–4 mL of BM-MSCs + PRP or 1–2 mL of AD-MSCs + PRP was injected intra-articularly into the stifle (volume depended on the dog’s size)	Neither treatment was superior to the other in terms of outcome (90 days).	[94]

**Table 4:** Summary of intervertebral disk degeneration (IVDD) disorders treated with MSCs.

Disease	Animal	Treatment	Route and dosage	Outcome	Ref.
IVDD	Dogs (n = 6)	Autologous BM-MSCs	Intradiscal injection of 2 × 10^6^ BM-MSCs suspended in 1 mL 10% autologous plasma in PBS. Only PSB solution was used for group control	Twelve months after treatment: even if the injection was well tolerated with no side effects, no successful treatment was found in any dogs.	[98]
IVDD- lumbosacral	Dogs (n = 20)	Autologous BM-MSCs	Intradiscal injection of 3 × 10^6^ was applied in three different groups—(1) intradiscal injection of MSC-microcarriers (n = 11), (2) MSC-TGF-β1-microcarriers (n = 6), and (3) microcarriers only during a decompressing spinal surgery (n = 3)	Ten months after treatment: injection was successful in all dogs; thus, they returned to normality. Schmorl’s nodes were found as side effects.	[99]
IVDD-chronic spinal cord injury	Dogs (n = 7)	Allogeneic foetal BM-MSCs	Intramedullary injection of 1 × 10^6^ allogeneic BM-MSCs	Ninety days evaluation showed no side effects, increased movement of the hind limbs, and increased locomotory function.	[100]
IVDD- Thoracolumbar intervertebral disc disease	Dogs (n = 34)	Allogeneic AD-MSCs + surgery	Intraoperative intraspinal allogeneic AD-MSCs of 1 × 10^7^ cells	Six months after treatment: Improvement in the neurological exam and better endpoint with AD-MSCs application rather than surgery only.	[101]
